# Obturator Nerve Block: An Anatomical Perspective

**DOI:** 10.7759/cureus.59125

**Published:** 2024-04-27

**Authors:** Rajiv Ranjan, Rita Kumari, Babita Kujur, Rana Pratap Singh, Aradhana Sanga

**Affiliations:** 1 Anatomy, Rajendra Institute of Medical Sciences, Ranchi, IND; 2 Urology, Rajendra Institute of Medical Sciences, Ranchi, IND

**Keywords:** clinical practice, nerve block, cadaveric dissection, anatomical localization, obturator nerve

## Abstract

Background

A comprehensive understanding of the anatomy of the obturator nerve after its emergence from the obturator foramen is essential when undertaking an obturator nerve block effectively. This study was conducted to provide precise anatomical guidance of the obturator nerve block with surface landmarks in the inguinal region.

Materials and methods

A cross-sectional observational study was carried out on 34 dissected embalmed cadaveric lower limbs to investigate anatomic variability of obturator nerve localization concerning bony/ligamentous landmarks viz. the pubic tubercle, anterior superior iliac spine, inguinal ligament, and femoral artery as well as the adductor longus.

Results

The pubic tubercle and inguinal ligament were found to be the "least variable indicator" and palpable landmark for localization of the main trunk of the obturator nerve exhibiting lesser standard deviation of the mean distance from the obturator nerve exit. Among the soft tissue (vessel/muscle) parameters, the shortest distance of the adductor longus muscle from the obturator nerve exit was found to have the lowest standard deviation, thus making it the most reliable parameter for obturator nerve localization.

Conclusion

High anatomic variability in the obturator nerve's localization does exist, and this explains the difficulty frequently encountered in the application of regional anesthetic techniques. The pubic tubercle and inguinal ligament points were found to be the least variable and most reliable landmarks for localization of the main trunk of the obturator nerve.

## Introduction

Obturator nerve block (ONB) is frequently used during transurethral excision of bladder tumors to avoid abrupt thigh adduction [1]. Additionally, this preferred local block is utilized to treat chronic hip pain, reduce persistent hip adductor spasticity in individuals with cerebral palsy, multiple sclerosis, or paraplegia, and give the best analgesia for knee surgery [2-8]. Based on surface landmarks, before the development of nerve stimulation, the classical ONB was first described by Labat in 1922 and detailed a paresthesia technique. The patient was supine during the technique, with the ipsilateral leg abducted at a 30-degree angle [9]. Since then, several ONB techniques have been documented that use surface features to locate the nerve, either with or without nerve stimulation [10-12].

The obturator nerve is a branch of the lumbar plexus formed within the substance of the psoas major from the ventral branches of the second to fourth lumbar ventral rami. As the nerve of the adductor compartment of the thigh, it reaches by piercing the medial border of psoas major and traveling straight along the lateral pelvic wall to the obturator foramen. From there, it crosses the medial pelvic brim to the sacroiliac joint and descends forward between the internal iliac vessels and the fascia on the obturator internus [13].

Here, the obturator nerve passes in close proximity to the inferolateral bladder wall. The obturator nerve enters the obturator foramen along the obturator canal with the obturator vessels lying anterosuperior to it. It is divided into anterior and posterior branches upon exiting from the obturator canal, first by the obturator externus and then by the adductor brevis more inferiorly. The thick fascia between the pectineus and obturator externus muscles contains the obturator nerve [14].

Rationale and objective of the study

There is a paucity of literature related to the exact localization of the obturator nerve concerning clear surface anatomical landmarks for localization of the obturator nerve, leading to complexity of the ONB and inconsistent results in the procedure, leading to infrequent use of the ONB. 

Our objective was to identify the anatomical landmarks for localization of the obturator nerve after its exit from the obturator foramen and to take the measurement of the relevant dimension for nerve block precision. Subsequently, we also studied the variation in course and branching of the extrapelvic part of the nerve.

## Materials and methods

A cross-sectional observational study was carried out using a convenience sampling method after obtaining Institutional Ethial Clearance (via letter no 378 dated 20/10/2021), on 17 (14 male and 3 female) formalin-embalmed adult human cadavers of North-Indian ethnicity aged between 20 to 65 years. The cadavers with anomalies related to the lower limb or pelvis and/or manifesting pelvic/perineal surgical procedures were excluded.

Data measurement

**Figure 1:**
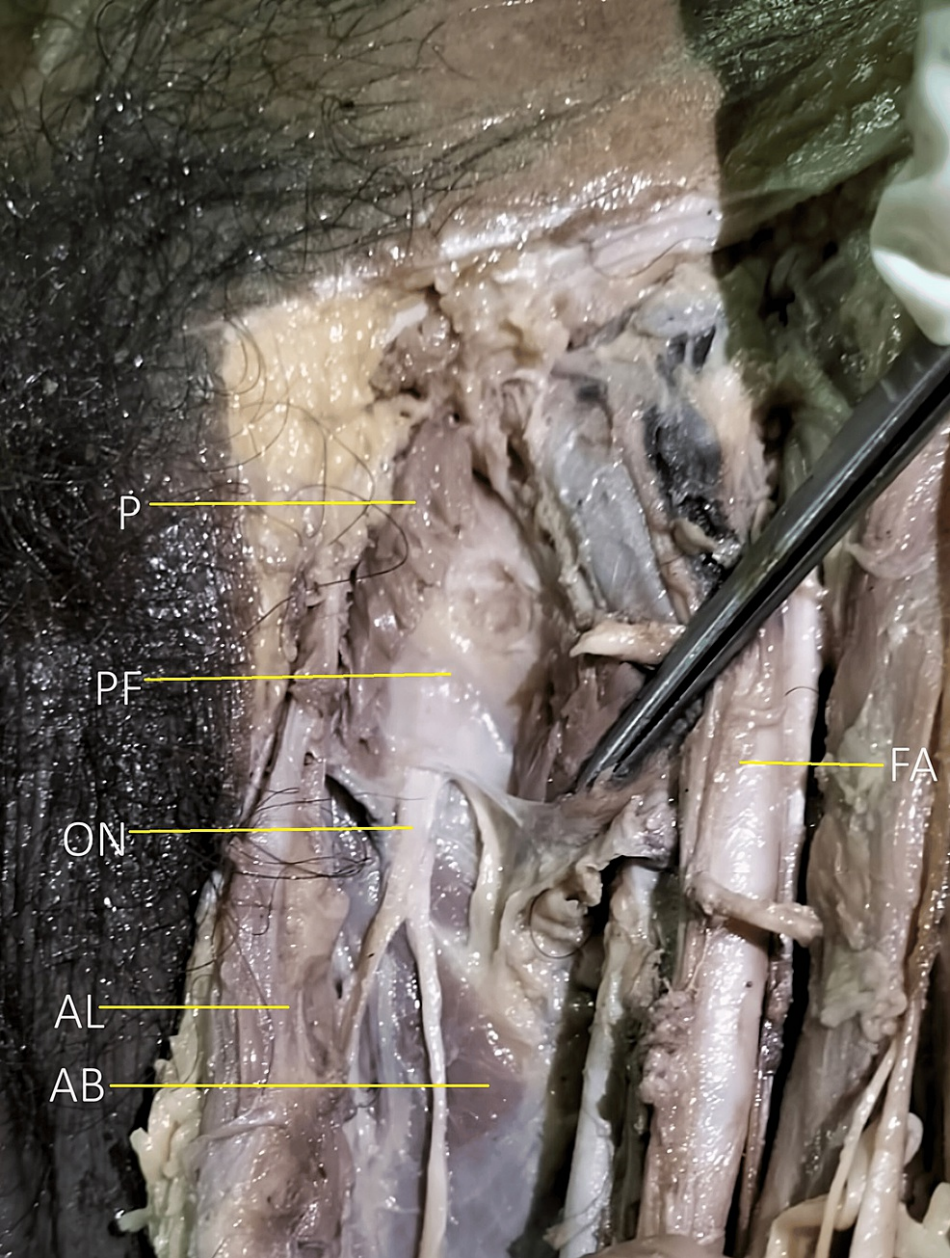
Left antero-medial aspect of the thigh of a male cadaver showing a consistent thick pectineal fascia (PF) overlying the obturator nerve emergence (ONE) Image showing a thick facia (PF) underneath the pectineus (cut margin marked as P) overlying the obturator nerve (ON) main trunk emergence. AL: Adductor longus, AB: Adductor brevis, FA: Femoral Artery.

Meticulous dissection of the adductor compartment was done as per Cunningham’s Manual of Practical Anatomy [15]. The obturator nerve was exposed through a ventral dissection approach with the cadaver in a supine position. The skin and fascia between the anterior superior iliac spine to the pubic tubercle superiorly and the medial and lateral condyle of the femur inferiorly was removed. Muscles of the adductor compartment were identified and its neurovascularity was noted. Thus, the obturator nerve was cleaned and traced. The emergence point of the nerve from the obturator foramina was cleaned after cutting the pectineus from the pectineal line. Over this, a thick pectineal fascia (PF) was consistently visible as an anatomical landmark overlying the main trunk of the obturator nerve and its division/branches in the vicinity. The picture below shows a thick facia (PF) underneath the pectineus (cut margin marked as P) overlying the obturator nerve (ON) main trunk emergence (Figure 1).

Thereafter, the adductor longus muscle inferior to its attachment on the inferior pubic ramus was cut. The main trunk and its branching pattern with variation were noted and photographed.

The below-mentioned strategic anatomical landmarks viz. palpable bony/ligamentous, vascular, and muscular were palpated/cleaned, and their dimensions from the obturator nerve trunk before bifurcation were measured with a digital Vernier caliper of ±0.02 mm accuracy.

Parameters measured

The depth (obturator nerve emergence (ONE) - pubic tubercle (PT) Vertical (V)) and horizontal dimensions (ONE - PT Horizontal (H)) of the emergence point of the obturator nerve from the pubic tubercle (AC) were measured. Initially, using a leveler, a hypodermic needle was placed at the ONE and adjusted at the same level as the upper margin of PT. The first measurement, the depth of ONE, was thus measured (ONE - PT V) using the hypodermic needle. Then, the second measurement (ONE - PT H) was taken as the distance between the needle and the upper margin of PT.

**Figure 2:**
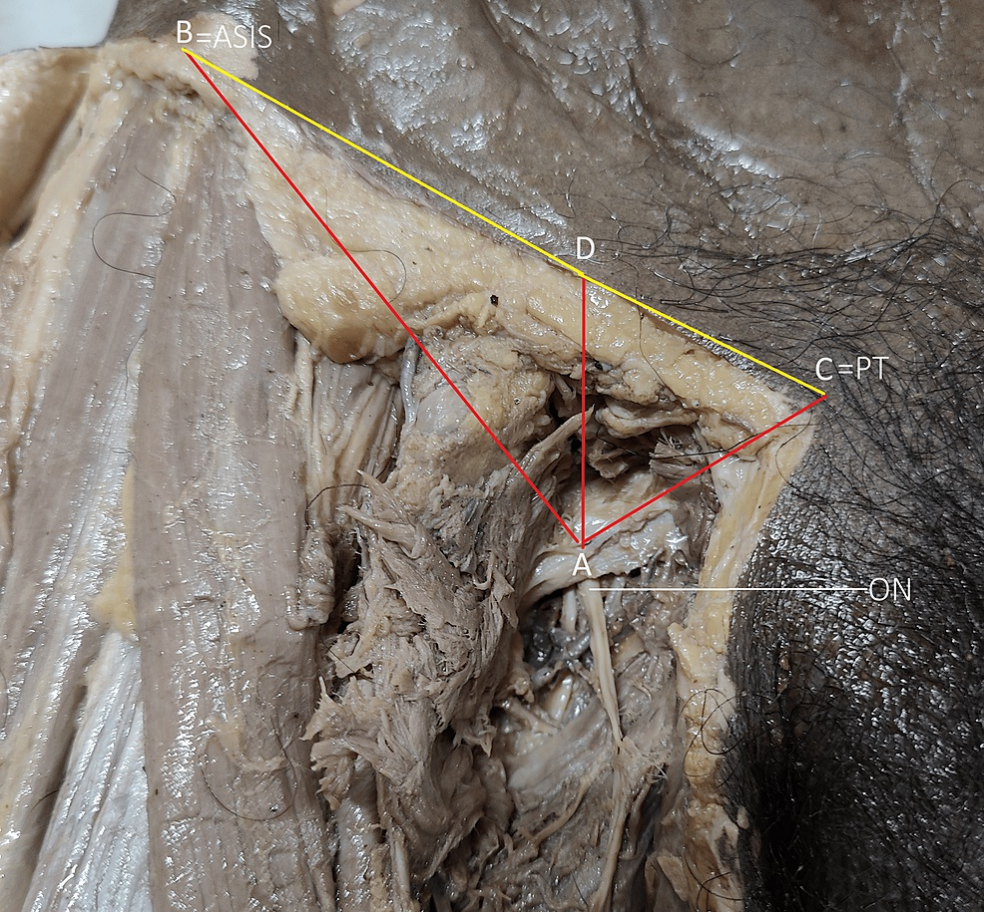
Schematic of dimensions measured between obturator nerve emergence (ONE) to various bony/ligamentous landmarks Various strategic bony/ligamentous dimensions (pubic tubercle-PT, Anterior superior illiac spine -ASIS) measured from obturator nerve (ON) emergence (ONE) ONE to ASIS:AB, ONE to PT:AC, ONE to Inguinal ligament:AD.

Other parameters measured were the distance of the emergence point of the obturator nerve from the pubic tubercle (PT) defined as AC, distance of the emergence point of the obturator nerve from the anterior superior iliac spine (ASIS) defined as AB, the vertical reference distance of the emergence point on the inguinal ligament (IL) defined as AD, distance of the inguinal ligament point (IL point) from the PT defined as DC, and distance of IL point to ASIS defined as DB (Figure 2).

The descriptive data obtained were analyzed and interpreted using the Jamovi software for Windows, Version 2.2.5.0 [16]. An independent sample t-test was conducted between gender, side of the limb, and different dimensions of the obturator nerve and important anatomical landmarks. We assumed equality of variance because the Levene's Test for Equality of Variances indicated a value of >0.05 for all variables. A p-value was less than 0.05 was considered statistically significant.

**Figure 3:**
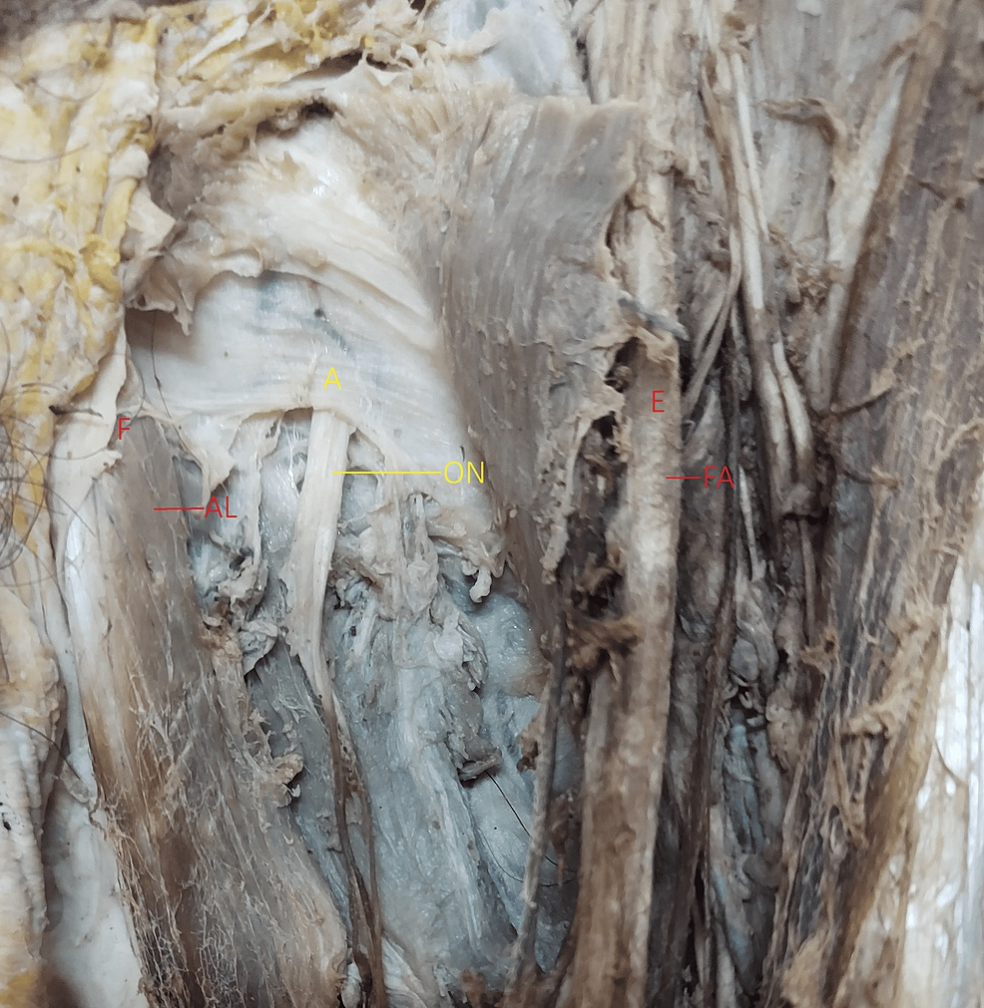
Schematic of dimensions measured between obturator nerve emergence (ONE) to the femoral artery and adductor longus The dimension of ONE to the medial margin of the femoral Artery (AE) and ONE to the lateral margin of the adductor longus (AF). Obturator nerve: ON, Femoral Artery: FA, Adductor longus: AL.

Additionally, the shortest distance of femoral artery from the obturator nerve emergence (AE), shortest distance of superior attachment of the lateral border of the adductor longus on the inferior pubic ramus from the obturator nerve emergence point (AF) was also measured (Figure 3).

## Results

In 17 cadavers (34 hemipelves), the localization of the obturator nerve from various anatomical landmarks was measured (n = 34), of which 3 were female and 14 were male. The cadaver's mean age was 53.88 ±11.39 years, with minimum and maximum ages of approximately 30 and 70 years, respectively.

The thickness of the obturator nerve was measured in 29 cases where the nerve divided after emergence and 5 remained unmeasured since the obturator nerve bifurcated into the anterior and posterior division before the exit (intrapelvic bifurcation). The minimum and maximum thickness of the nerve’s main trunk was 2 and 7 mm, respectively, and its mean± standard deviation was 3.86±1.24 mm. A statistical analysis of the anatomical landmarks of the obturator nerve was performed. The Shapiro-Wilk test showed the various anatomical landmarks to be normally distributed (p-value >0.05) across the right and left sides (Table 1). An independent sample t-test was done to see the significant difference in the mean of laterality about anatomical landmarks. The absence of statistical significance on the two sides signifies that precise localisation and accurate block of obturator nerve can be done on the basis of anatomical landmarks equally on both sides. The reliable hard landmarks (bone/ligament) were the pubic tubercle (PT) and inguinal ligament (IL) points as they exhibit the lowest SD (Table 1).

**Table 1:** Localization of the obturator nerve with respect to anatomical landmarks across the right and left sides ON exit: obturator nerve exit, ONE: obturator nerve emergence, PT Horizontal: pubic tubercle Horizontal, PT Vertical: pubic tubercle vertical, IL: inguinal ligament, ASIS: anterior superior iliac spine, PT: pubic tubercle, FA: femoral artery, AL: adductor longus.

Serial.No	Variables	Laterality	Mean ± SD (in mm)	p-value	T value
1	ON Exit - PT Horizontal	Right	19.82 ± 5.00	0.082	1.798
		Left	17.12 ± 3.67		
2	ONE - PT Vertical	Right	28.47 ± 4.50	0.275	1.112
		Left	30.29 ± 5.04		
3	ONE - IL	Right	17.41 ± 6.32	0.412	.830
		Left	15.94 ± 3.64		
4	ASIS to IL point	Right	88.18 ± 11.63	0.435	-.790
		Left	91.12 ± 10.02		
5	PT to IL point	Right	22.12 ± 6.57	0.242	1.192
		Left	19.71 ± 5.13		
6	ONE- ASIS	Right	99.00 ± 10.75	0.851	-.190
		Left	99.65 ± 9.08		
7	ON Exit - FA	Right	24.35 ± 5.29	0.727	-.352
		Left	24.88 ± 3.23		
8	ON Exit - AL	Right	14.65 ± 4.83	0.503	.677
		Left	13.69 ± 3.04		

Among the soft tissue (vessel/muscle) landmarks, the distance of the adductor longus of the left side from the obturator nerve exit was found to have the lowest SD, while the distance of ASIS from the IL point was found to have the highest SD (Table 1).

The localization of the obturator nerve was statistically analyzed with respect to gender. The Shapiro-Wilk test showed the various anatomical landmarks to be normally distributed across genders. An independent sample t-test was used to determine whether there was a significant mean difference between gender with respect to anatomical landmarks. The mean difference between ONE to IL and ONE to AL was statistically significant (p≤0.05) among genders (Table 2).

**Table 2:** Localization of obturator nerve with respect to anatomical landmark among gender ON Exit: Obturator nerve exit, PT H: pubic tubercle Horizontal, PTV: pubic tubercle vertical, IL: inguinal ligament, ASIS: anterior superior iliac spine, PT: pubic tubercle, FA: femoral artery, AL: adductor longus.

S.No	Variables	Gender	Mean ± SD	p-value	T value
1	ONE - PT H	Male	18.29 ± 4.89	0.615	-.508
		Female	19.33 ±2.25		
2	ONE - PTV	Male	29.36 ± 5.20	0.948	-.065
		Female	29.50 ± 2.34		
3	ON Exit - IL	Male	17.54 ± 5.13	0.033	2.229
		Female	12.67 ±2.87		
4	ASIS to IL point	Male	89.04 ±11.60	0.484	-.708
		Female	92.50 ±5.54		
5	PT to IL point	Male	20.57± 6.33	0.484	-.717
		Female	22.50 ±3.50		
6	ON Exit- ASIS	Male	99.61± 10.57	0 .722	.359
		Female	98.00± 5.29		
7	ONE - FA	Male	24.57 ±4.31	0.895	-.133
		Female	24.83± 4.79		
8	ONE - AL	Male	13.44± 3.75	0.023	2.385
		Female	17.50 ±3.83		

## Discussion

The objective of the current study was to obtain a through understanding of the obturator nerve's surface anatomical location in embalmed cadavers, with implications for directing obturator nerve blocks in clinical settings. The most reliable bone/ligament landmarks for ONB in limited resource clinical settings are the PT and IL points with lesser standard deviation of the mean distance from the obturator nerve emergence. Alternatively, among the soft tissue (vessel/muscle) landmarks for ultrasound-guided effective ONB are the distance of the adductor longus from the obturator nerve emergence was found to have the least standard deviation, thus making it the most reliable parameter for obturator nerve localization. The least reliable parameter was the distance of ASIS from the IL point having the highest standard deviation.

The shortest distance of the ONE to the IL was found to be a point located at the junction of the medial 1/4th and lateral 3/4th of a line joining ASIS to PT. A comparison of the right and left sides shows that all the parameters measured from the ONE were statistically non-significant (P ≥ 0.05). The absence of statistical significance on the two sides signifies that precise localisation and accurate block of obturator nerve can be done on the basis of anatomical landmarks equally on both sides. The precise location of the nerve trunk could be located at 16.66 ± 4.98 mm below the point located at the junction of the medial 1/4th and lateral 3/4th of a line joining ASIS to PT. 

Comparison with existing literature

Our observations aligned with previous anatomical studies that have emphasized the variations in the obturator nerve's course and relationships. The findings of Jo et al. in 14 embalmed cadavers of Korean origin are similar to our findings, thus reiterating the role of these anatomical landmarks in cadavers of Indian origin. [17](Table 3)

**Table 3:** Comparison of findings reported in Jo et al. and the present study. ON Exit: obturator nerve exit, IL: inguinal ligament, ASIS: anterior superior iliac spine, PT: pubic tubercle, FA: femoral artery, AL: adductor longus.

Parameter	Jo et al. [17]	Present study
Sample size	14 cadavers	17 cadavers
ONE to ASIS (Right vs Left)	113.4±6.5mm (R) 114.2±7.4mm (L)	99.00 ± 10.75(R) 99.65 ± 9.08(L)
ONE to PT (Right vs Left)	18.2±3.2mm (R) 16.5±3.0mm (L)	19.82 ± 5.00(R) 17.12 ± 3.67(L)
ONE to IL (Right vs Left)	18.5±3.2mm (R) 19.7±5.2mm (L)	17.41 ± 6.32(R) 15.94 ± 3.64(L)
ONE to FA (Right vs Left)	30.0±5.4mm (R) 25.8±3.9mm (L)	24.35 ± 5.29(R) 24.88 ± 3.23(L)

In their study on 67 lower limbs of the Ethiopian population, Berhanu et al. reported that the bifurcation levels of the obturator nerve were intrapelvic in 23.9%, within the obturator canal in 44.8%, and extrapelvic 31.3% [18]. In the study on 84 dissected embalmed cadavers, Anagnostopoulou et al. reported that division of the obturator trunk was intrapelvic in 23.22%, within the obturator canal in 51.78%, and in the thigh in 25%. The fascia medial to the femoral vessels and deep to the pectineus muscle were visualized (100%) by ultrasound imaging [19]. The thickness of the obturator nerve (mm) was measured in 29 cases and remained unmeasured in the remaining 5 since the obturator nerve bifurcated into the anterior and posterior division before the exit. Thus, the percentage of intrapelvic bifurcation in our study was found to be 14.70%. Our study builds upon this foundation by providing precise measurements and visual documentation, allowing for a more comprehensive understanding of the nerve's anatomical nuances.

Clinical implications

Accurate localization of the obturator nerve is paramount for the success of obturator nerve blocks. Our detailed anatomical insights offer valuable guidance to the anaesthetist and surgeons performing these procedures. By providing measurements of distances between the nerve and adjacent landmarks, our study contributes to improved needle placement accuracy and reduces the risk of complications such as nerve injury.

Understanding the precise anatomical location of the obturator nerve is crucial for medical educators guiding future practitioners in nerve block procedures. Our insights can be used to create anatomical guides that will improve training programs by giving students access to trustworthy anatomical references.

Limitations

Although dissections of cadavers offer insightful information about anatomy, post-mortem changes and embalming with 10% formalin can cause shrinkage and modify the properties of the tissue. Furthermore, the generalizability of our findings may be constrained by the limited sample size and cadaver demographics. To validate our findings, future research must concentrate on extending the study cohort to include a wider variety of cadavers and using complementary imaging methods like ultrasonography along with contrast media inoculation. Studies with a longitudinal design could evaluate how stable the observed anatomical correlations are over time. An inherent limitation of this study is the disproportionate gender distribution among cadaver specimens, with a ratio of 3:14 female to male. While efforts were made to obtain a diverse sample, the skewed gender distribution may influence the generalizability of our findings to broader populations. Future studies with a more balanced gender representation are warranted to further validate our findings.

## Conclusions

To conclude, our research sheds light on the precise anatomical location of the obturator nerve in embalmed cadavers. Its clinical relevance as a guide for obturator nerve blocks is improved by thorough visualization, measurements from surface anatomical landmarks, and documentation of the nerve's course and relationships. While real-time imaging benefits are provided by ultrasound-guided techniques, cadaveric dissection offers a deeper understanding of anatomical variations and spatial relationships that may enhance the safety, accuracy, and effectiveness of obturator nerve blocks in clinical practice without involving live subjects and the associated ethical issues. Integrating both approaches can offer a comprehensive understanding of optimized procedural outcomes, which ultimately benefits patients and surgeons alike by bridging the gap between anatomical knowledge and clinical practice.
